# Socio-economic burden of TB and its impact on child contacts in The Gambia

**DOI:** 10.5588/pha.23.0025

**Published:** 2023-12-07

**Authors:** A. K. Sillah, I. Devoid, J. J. Ndenkeh, G. Moonga, I. Loum, A. Touray, O. Owolabi, J. Sutherland, A. Rachow, O. Ivanova, D. Evans, B. Kampmann

**Affiliations:** 1 Vaccines & Immunity Theme, Medical Research Council Unit The Gambia at the London School of Hygiene & Tropical Medicine, Banjul, The Gambia; 2 Center for International Health, University Hospital, Ludwig Maximilian University of Munich, Munich, Germany; 3 Faculty of Public Health and Policy, London School of Hygiene & Tropical Medicine, London, UK; 4 Division of Infectious Diseases and Tropical Medicine, Medical Centre of the University of Munich (LMU), Munich, Germany; 5 German Centre for Infection Research (DZIF), Partner Site Munich, Munich, Germany; 6 Health Economics and Epidemiology Research Office, Faculty of Health Sciences, University of the Witwatersrand, Johannesburg, South Africa; 7 Charité Centre for Global Health, Charité Universitatsmedizin-Berlin, Berlin, Germany

**Keywords:** tuberculosis, socio-economic impact on children, household contacts;

## Abstract

**OBJECTIVE:**

To determine the social impact of adult TB on child household contacts living in the Greater Banjul Area, The Gambia.

**METHODS:**

This was a prospective observational cohort study among adults (≥18 years) starting treatment for drug-susceptible pulmonary TB between June 2019 and July 2021 who reported having at least one child household contact. We collected data from 51 adults and 180 child contacts at the start of TB treatment (baseline) and again at 6 months of treatment. Participants were asked about expenses for school fees, healthcare, festivities and food security of child contacts.

**RESULTS:**

While school attendance of the child contacts remained largely unaffected, there was a significant drop in school performance at 6 months (*P* < 0.001). Furthermore, child contacts faced significant food insecurity in terms of food quantity and variety available, with up to a four-fold increase in some instances at 6 months compared to baseline (*P* < 0.001)

**CONCLUSION:**

Child contacts face a potential decline in school performance and risk of food insecurity. While a plethora of work is being undertaken to alleviate costs of care for TB patients, further emphasis is needed to ensure educational and social prosperity for child contacts, as adults with TB have socio-economic implications for the wider household.

## Abstract (fr)

**OBJECTIF:**

Déterminer l’impact social de la TB de l’adulte sur les contacts familiaux de l’enfant vivant dans la région du Grand Banjul, en Gambie.

**MÉTHODES:**

Il s’agissait d’une étude de cohorte observationnelle prospective auprès d’adultes (≥18 ans) commençant un traitement contre la TB pulmonaire sensible aux médicaments entre juin 2019 et juillet 2021 et qui ont déclaré avoir au moins un contact domestique avec un enfant. Nous avons recueilli des données auprès de 51 adultes et 180 enfants contacts au début du traitement contre la TB, puis à nouveau après 6 mois de traitement. Les participants ont été interrogés sur les dépenses liées aux frais de scolarité, aux soins de santé, aux célébrations et à la sécurité alimentaire des enfants contacts.

**RÉSULTATS:**

Alors que la fréquentation scolaire des enfants contacts n’a pratiquement pas été affectée, on a constaté une baisse significative des résultats scolaires 6 mois plus tard (*P* < 0,001). Par ailleurs, les enfants contacts ont été confrontés à une insécurité alimentaire importante en termes de quantité et de variété de nourriture disponible, avec une augmentation d’au moins quatre fois après le traitement de la TB (*P* < 0,001).

**CONCLUSION:**

Les enfants contacts sont confrontés à une baisse potentielle de leurs résultats scolaires et à un risque d’insécurité alimentaire. Alors qu’une multitude de travaux sont entrepris pour réduire les coûts des soins pour les patients atteints de TB, il est nécessaire de mettre davantage l’accent sur la prospérité éducative et sociale des enfants contacts, étant donné que les adultes atteints de TB ont des implications socio-économiques pour l’ensemble du ménage.

While the COVID-19 pandemic has strained health systems across the world, TB has contributed substantially to the global burden of disease,^1^ with significant impact on childhood TB management.^2^ Many patients suffer long-term consequences due to pulmonary TB (PTB) even after completion of treatment and complete bacteriological cure,^3^ and many patients face economic and social consequences that can have implications on their households, including children.^4^

There is a growing consensus among governments, researchers and non-governmental organisations that progress to control TB in low- and middle-income countries (LMICs) will require both the strengthening TB control programmes, as well as an emphasis on the social determinants of health.^5^,^6^ Attention on the social determinants of health is partly due to a growing body of literature documenting the association between substantial TB patient costs that can cause or exacerbate poverty and negatively impact TB treatment outcomes and adherence.^7^ Such literature has influenced a variety of social protection schemes across LMICs to enable patients to complete treatment and limit the likelihood of financial catastrophe for their households. In The Gambia, TB treatment is provided free at the point of access across governmental facilities; however, patients can still incur substantial non-medical direct (e.g., transport, food, care provided by family) and indirect costs (i.e., loss of income) in accessing treatment.^8^ While tools to assess the household economic burden of TB, such as the WHO’s generic TB patient cost survey contains questions on household food security, the instrument does not capture the full spectrum of social and economic consequences that children of adults with TB may encounter.^9^ While research on the social and economic consequence of adult TB in children is limited, a study in India by Geetharamani et al. showed that TB infection in adults can result in declining school performance in children, with some children leaving school entirely to support their households financially.^10^

While it may already be difficult for households to recover from the economic shocks of TB disease, school performance, dropout and food security can have implications on children that last into adulthood.^11^,^12^ While research focusing on TB patient costs has been paramount in designing social protection schemes, it is imperative that research addresses in what way children are impacted to ensure that future policies can efficaciously protect this vulnerable population. Therefore, this study aims to explore the social and economic consequences of household TB on children in The Gambia, a country with a relatively low incidence of TB, but which is among the poorest in the world.

## METHODS

### Study setting

The Gambia, a small country in West Africa, has a relatively low prevalence of TB with an incidence rate of 149 cases per 100,000 population.^13^ Two-thirds of TB patients are diagnosed in the Greater Banjul Area (GBA), which has a population of nearly 700,000 inhabitants and spans Banjul, Kanifing Municipality (KM) and parts of Brikama Administrative Area.^14^

### Study participants and data collection

This prospective observational study was based on data collected at the Medical Research Council Unit The Gambia at the London School of Hygiene & Tropical Medicine (MRCG at LSHTM) in the GBA. Longitudinal collection of socio-economic data was nested in the large multi-country, multi-site TB Sequel study (NCT03251196). Adults (≥18 years) with pulmonary TB (PTB) were enrolled at TB treatment initiation between September 2017 and January 2020, and received TB treatment according to the local standard of care. Participants were followed for up to 48 months, with interviews conducted at study enrolment (Day 0 ±14 days), 2, 6, 12, 24, 36 and 48 months.^3^

Informed consent was obtained from eligible participants. Demographic information, including site of disease, age, sex, ethnicity, marital status and HIV status, was collected from longitudinal surveys at baseline (start of treatment). Questionnaires were administered in English and, where necessary, translated into the local languages in the study clinics or at the homes of study participants by trained interviewers.

For this country-specific study, TB Sequel participants enrolled between June 2019 and July 2021 reporting having at least one child household contact (children <15 years of age) were identified. Eligible participants provided written informed consent to participate and were interviewed at baseline and at 6 months. We defined a household contact as a person who shared the same enclosed living space as the index case for one or more nights or for frequent or extended daytime periods.^15^ The interview included questions on sociodemographic and household information such as employment status, education, family type, size of family, number of child contacts and their age ranges, child contacts’ education such as school attendance, school performance and other educational needs for both integrated preschool (3–5 years) and school-aged children (5–15 years). Data on school attendance and school performance were extracted from school report cards.

We collected information on spending for items such as clothing, shoes and gifts during feast celebrations (when parents buy new clothes, shoes and gifts for the family for celebrating such events) and on direct out-of-pocket expenses (medical and non-medical direct costs) for accessing healthcare for sick household child contacts, as well as on school essentials (e.g., uniform, school fees, extra classes, meals, transportation and stationery). The recall period for reporting on socio-economic and cost data was 4 months. We collected information on the ability of the adult TB parent to provide enough quality food before and during TB illness for the child contacts. Data on household income and costs incurred were drawn from patient recall and not extracted from pay stubs or receipts.

### Data analysis

Analysis was done using SPSS software (IBM Corp, Armonk, NY, USA) and MS Excel (Microsoft, Redmond, WA, USA). First, to describe the characteristics of adult participants at baseline (start of TB treatment) and their child contacts, we summarise data using means (standard deviation [SD]) for parametric or median (interquartile range [IQR]) for non-parametric continuous data and proportions for categorical data. We only collected the age, and not the sex of child contacts (as we did not segregate our childhood participants by sex in our study questionnaire, this cannot be factored in the present analysis, but will be factored in our broader study). The Figure shows the relationship between the various characteristics, TB treatment and impact on household child contacts.

To describe the economic status and participant’s ability to spend money on household child contacts, we present the change in employment, social support received (monetary and non-monetary), direct out-of-pocket expenses on healthcare for sick contacts and buying power from baseline to 6 months on treatment. Categorical questions were transformed into grades, and these were compared at baseline and 6 months on treatment using test for symmetry, χ^2^ test and McNemar’s test for categorical variables, where appropriate. Continuous variables were compared using the Wilcoxon sign-rank test (two groups; paired data). Comparative analyses were conducted where data were available at both baseline and 6 months, with a significance level of 0.05.

Using the same approach described above, we describe the impact on adult TB on school performance, school attendance, spending on child or school needs and child nutrition in terms of provision of types and quantity of food.

### Ethical considerations

Local ethics approval was obtained from The Gambia Government/MRCG at LSHTM Joint Ethics Committee, Banjul, The Gambia (SCC1636). The study also received ethics approval from Ludwig Maximilian University of Munich (LMU) Ethics Commission, Munich, Germany (Projekt Nr: 19-481). Informed consent was provided by all participants.

## RESULTS

### Characteristics of adult TB patients and their child contacts

Table 1 shows the baseline demographic characteristics of adult patients with PTB and at least one child household contact. A total of 53 patients (66.7% male, mean age: 39 years, SD 11.0) with child contacts were enrolled in this country-specific study. One participant died before the 6 months’ visit and another participant declined further participation; therefore, a total of 51 adults and 180 child contacts were included, providing an average of 3.5 child contacts per adult index case.

**TABLE 1:** Characteristics of adult participants at the start of TB treatment (baseline) and their child contacts

Variables	Total (*n* = 51) *n* (%)	Male (*n* = 34) *n* (%)	Female (*n* = 17) *n* (%)
Adult characteristics			
Age, years, mean ± SD	39.0 ± 11.0	40.7 ± 10.7	35.5 ± 11.1
18–30	14 (27.4)	6 (17.7)	8 (47.1)
31–40	16 (31.4)	12 (35.3)	4 (23.3)
>40	21 (41.2)	16 (47.1)	5 (29.4)
Marital status			
Single	6 (11.5)	4 (11.4)	2 (11.8)
Married/have partner	41 (78.8)	30 (85.7)	11 (64.7)
Divorced	3 (5.8)	1 (2.9)	2 (11.8)
Widowed	2 (3.8)	0	2 (11.8)
Education			
No formal education	3 (5.9)	1 (2.9)	2 (11.8)
Primary education	6 (11.8)	3 (8.8)	3 (17.7)
Arabic/Dara	21 (41.2)	12 (35.3)	9 (52.9)
Secondary education	18 (35.3)	16 (47.1)	2 (11.8)
University/vocational	3 (5.9)	2 (5.9)	1 (5.9)
Primary income earner in the household	27 (52.9)	23 (85.2)	4 (14.8)
Type of family			
Single parent family	8 (15.7)	3 (8.8)	5 (29.4)
Nuclear family	19 (37.3)	19 (55.9)	0
Extended family	24 (47.1)	12 (35.3)	12 (70.6)
Family size (number of children)			
1–3	28 (54.9)	18 (52.9)	10 (58.8)
4–5	13 (25.5)	9 (26.5)	4 (23.5)
≥6	10 (19.6)	7 (20.6)	3 (17.7)
Child contacts characteristics	Total (*n* = 180)		
Age, years			
0–5	63 (35.0)		
6–10	68 (37.8)		
11–15	49 (27.2)		

SD = standard deviation.

### Economic status and participant’s ability to spend money on household child contacts

Table 2 shows the proportion of participants receiving childcare or financial support at 6 months on TB treatment was almost double that reported at baseline, both of which were statistically significant (*P* = 0.037 and *P* = 0.027, respectively). Compared to baseline, there was strong evidence of a decrease in buying power at 6 months on TB treatment for clothes and shoes for feasts (e.g., during Muslim Eids and Christmas celebrations) from 96.1% (49/51) and 94.1% (48/51) to 64.0% (32/51) and 60% (30/51), respectively (*P* < 0.001 and *P* < 0.001, respectively). These were also accompanied by strong evidence of a decrease in the monetary sum used to buy clothes or shoes for feasts at 6 months on treatment, compared to baseline (*P* = 0.006 and *P* = 0.014, respectively).

**TABLE 2:** Economic status and participant’s ability to spend money on household child contacts

Indicator	Baseline *n* (%)	6 months *n* (%)	*P* value
Employment			
Unemployed	6 (12)	4 (8)	0.270^*^
Student	5 (10)	3 (6)	
Employed: informal	24 (48)	25 (50)	
Employed: formal	15 (30)	18 (36)	
Social support			
Childcare support received			0.037^†^
No	42 (82.4)	32 (64.0)	
Yes	9 (17.6)	18 (36.0)	
Financial support received			0.027^†‡^
No	39 (78.0)	28 (57.1)	
Yes	11 (22.0)	21 (42.9)	
If ‘yes’, frequency of financial support			0.452^‡^
Yearly	0 (0)	1 (5.3)	
Quarterly	1 (9.1)	4 (21.1)	
Monthly	5 (45.5)	4 (21.1)	
On demand	5 (45.5)	10 (52.6)	
Direct costs on healthcare, GMD, median [IQR]			
Cost of medical care for sick contacts	175 [75–225]	125 [0–250]	0.655^§^
Cost of non-medical care for sick contact	42 [0–110]	65 [50–80]	0.655^§^
Total healthcare expenditure	175 [117–335]	190 [80–300]	0.180^§^
Buying power, GMD, median [IQR]			
Bought clothes for feast	49 (96.1)	32 (64.0)	<0.001^†‡^
If yes, amount	2,500 [1,000–4,700]	2,000 [700–3,500]	0.006^†§^
Bought shoes for feast	48 (94.1)	30 (60.0)	<0.001^†‡^
If yes, amount	750 [400–1,000]	425 [300–888]	0.014^†§^
Bought items/gifts for feast	15 (29.4)	8 (16.3)	0.120^‡^
If yes, amount	200 [100–400]	80 [15–250]	0.777^§^

*Test of symmetry.

^†^Statistically significant.

^‡^χ^2^ test.

^§^Wilcoxon signed rank test.

GMD = Gambian dalasi; IQR = interquartile range.

### The impact of adult TB on the education and nutrition of child contacts

Table 3 shows that school attendance slightly declined; however, there was strong evidence of a decline in the academic performance of child contacts (end of term examination scores) with 7.3% (3/41) performing very well at 6-month TB follow-up compared with 51.2% (21/41) at baseline (*P* < 0.001).

**TABLE 3:** Impact of adult TB on the education and nutrition of child contacts

Indicator	Baseline *n* (%)	6 months *n* (%)	*P*-value
School attendance			0.220^*^
Regular school attendance	42 (87.5)	38 (79.2)	
Inconsistent school attendance	6 (12.5)	10 (20.8)	
School performance			<0.001^†^^‡^
Average (scores: 40–54%)	3 (7.3)	9 (22.0)	
Above average (scores: 55–64%)	0 (0)	10 (24.4)	
Good (scores: 65–74%)	17 (41.5)	19 (46.3)	
Very good (scores: 75–84%)	21 (51.2)	3 (7.3)	
School expenditure, GMD, median [IQR]			
School fees	2,000 [3,500–1,500]	1,200 [2,400–1,100]	0.003^†^^§^
Uniform	1,500 [2,345–675]	950 [1,500–500]	<0.001^†^^§^
Physical education set	438 [600–400]	275 [575–100]	0.162^§^
Extra classes	575 [1,400–250]	675 [1,175–250]	0.042^†^^§^
School shoes	750 [1,200–500]	550 [850–300]	0.004^†^^§^
Prepare school feeding	3,000 [4,000–700]	1,000 [3,200–400]	0.153^R^
Expenditure on food supplies at school	70 [20–225]	30 [20–58]	<0.001^†^^§^
Stationary	988 [1,473–500]	700 [1,500–450]	0.021^†^^§^
Transportation	1,800 [3,600–1,600]	1,360 [2,340–900]	0.113^§^
Nutrition			
Concerns that contacts will not have enough food	5 (10.0)	28 (56.0)	<0.001^*^^†^
Contacts unable to eat preferred foods	5 (10.0)	20 (40.0)	0.0006^*^^†^
Contacts eat limited variety of foods	2 (4.0)	16 (32.0)	<0.001^*^^†^
Contacts eat unwanted types of food	3 (6.0)	14 (28.0)	<0.001^*^^†^
Contacts eat small quantity of food	2 (4.0)	8 (16.0)	<0.001^*^^†^
Food not available at all	2 (4.0)	4 (8.0)	0.500^*^
Contacts go to bed at night hungry	2 (3.9)	2 (3.9)	1.000^*^

*McNemar’s test.

^†^Statistically significant.

^‡^Test of symmetry.

^§^Wilcoxon signed rank test.

GMD = Gambian dalasi; IQR = interquartile range.

When we assessed the impact on the different school expenditures on the child contacts before and during TB illnesses, we found evidence of a decline in expenditures on school fees, uniforms, shoes, food supplies at school and stationaries for child contacts at 6-month follow-up for TB treatment compared to baseline (*P* ≤ 0.05). Conversely, there was evidence of an increase on expenditure on extra classes from Gambian dalasi (GMD) 575 (USD11.24; using the average 2020 exchange rate GMD51.135 = USD1) at baseline to GMD675 (USD13.20) at 6-month follow-up (*P* = 0.042).^16^

After 6 months of TB treatment follow-up, 56% (28/50) of participants were worried child contacts would not have enough food, 40% (20/50) where child contacts were unable to eat preferred foods, 32% (16/50) where child contacts ate limited variety of foods and 28% (14/50) where child contacts ate unwanted food types, all of which were significant increases compared to baseline (*P* < 0.001). Furthermore, there was evidence of an increase in the proportion of participants whose child contacts ate small quantities of food, from 4% (2/50) at baseline to 16% (8/50) at 6 months (*P* < 0.001).

## DISCUSSION

Our study reports on the economic and social consequences faced by childhood contacts of adult TB patients in The Gambia. Our results suggest that TB in adults is associated with a wide range of effects on the educational, social and nutritional components of childhood contacts’ lives. There was strong evidence of an increase in participants reporting household food insecurity, including child contacts having insufficient, unwanted and small proportions of food. Moreover, at 6 months’ on TB treatment, there was a decline in buying power for adults, as well as a decline in spending on school fees, stationary and school shoes.

To our knowledge, this is the first study to collect direct cost and school performance data from adults with TB pertaining to their child contacts; however, several studies have descriptively captured social factors of TB illness such as school dropout and food insecurity due to TB in an adult. Children in our study did not experience school dropouts or exhibit significant evidence of a decline in regular school attendance. However, in Geetharamani et al., Chand et al. and Honavar et al., respectively 11%, 12% and 8% of child contacts dropped out of school.^10^,^11^,^17^ The difference is plausibly due to study setting as the aforementioned studies all took place in non-African settings. Additionally, in our study there was substantial caring and financial support from the affected families that potentially enabled the children to remain in school. While school attendance remained consistent, factors of household food insecurity could have led to the decline in school performance for the children. Previous literature has exhibited an association between malnutrition in children with adult TB contacts. For example, in Geetharamani et al., 34% of adults reported not being able to buy adequate food for their children. In our study, there was a negative association between adult TB and measures of household food insecurity, suggesting that child contacts may have had inadequate food portions, as well as limited and unwanted varieties of foods.^10^ A plethora of research has found an association between food-insecure households and negative physical health outcomes like underweight, stunting and wasting.^1819^ In addition to physical health outcomes, there is a negative association between food-insecure households and academic and cognitive functions such as lower literacy, numeracy and short-term memory in children.^20^,^21^ It is thus plausible that the decrease in children’s post-TB illness school performance is associated with the increase in food-insecure households. Furthermore, the decline in spending on school-related supplies suggests that adults with TB likely do not have the monetary or physical or psychological wellbeing to support their child contacts in school through their TB illness.

While our study has immediate policy implications for The Gambia, they also warrant consideration for other countries, specifically LMICs. The decline in spending, household food security and school performance highlights the need for policy shifts to address the social and economic consequences that children living in households with TB face. Like many countries in sub-Saharan Africa, The Gambia provides free TB diagnosis and treatment at government facilities. However, a recent study from the same cohort of TB cases reported that patients can still incur catastrophic costs (i.e., costs that exceed 20% of household income), despite the provision of free treatment. Economic support such as food and transportation vouchers can reduce patient costs throughout the TB episode, preserving the household’s disposable income to spend on essentials such as food and school-related supplies.^8^ While engaging adult TB patients in care is especially important to reducing direct and indirect costs that patients, and ultimately, their families incur, future policy should address the secondary effects on children.

Cash transfer programmes have long been used to improve TB treatment retention and outcomes, and reduce the proportion of patients experiencing catastrophic costs.^22^ TB cash transfers are typically given to households with a confirmed TB case to enable access to treatment and nutritional support.^23^ However, there is evidence that conditional cash transfers can improve school attendance.^24^,^25^ Future policymakers could consider educational cash transfer programmes for poor families conditional on child contacts maintaining regular school attendance. Such an approach would incentivise school attendance for child contacts, while providing needed funds to address household food insecurity, allowing children to focus on their school studies.

While our research provides valuable information on school performance, spending on children and household food security, future research on child TB contacts should include specific healthcare measurements such as child malnutrition, wasting and mental health outcomes, as well as the impact of stigma on social networks and relationships.

Our study has limitations. As we collected costs and outcomes data from TB patients recruited at the MRCG at LSHTM and government health facilities, it is likely that the poorest TB patients who did not have the resources, were too ill to engage in formal care or died were excluded. Additionally, our results are prone to social desirability bias, as adults are likely to over report more desirable outcomes such as adequate funds to pay for supplies or foods, thus, potentially underestimating the proportion of food-insecure households and those able to buy basic school supplies.

## CONCLUSION

While there is a growing body of literature detailing the health and economic impacts that TB patients face, this is among the first studies to quantitively assess the impact on their children in sub-Saharan Africa. These children experienced decreases in school performance, and spending on school supplies, and declines in household food insecurity. Policy changes are needed to ensure that child contacts are not impeded in their growth and education.

**FIGURE:**
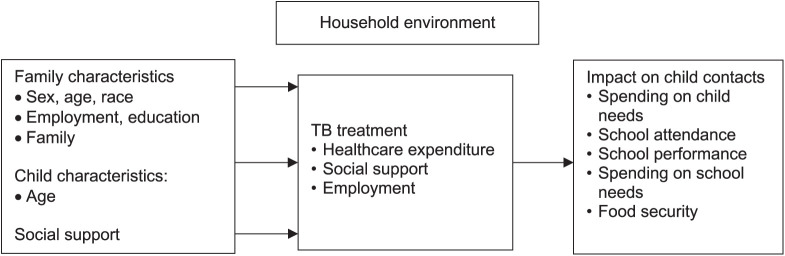
Graphical depiction to show the relationship between the various characteristics, TB treatment and impact on household child contacts.
